# Cdc6 disruption leads to centrosome abnormalities and chromosome instability in pancreatic cancer cells

**DOI:** 10.1038/s41598-020-73474-6

**Published:** 2020-10-05

**Authors:** Yuna Youn, Jong-chan Lee, Jaihwan Kim, Jae Hyeong Kim, Jin-Hyeok Hwang

**Affiliations:** 1 Department of Internal Medicine, Seoul National University Bundang Hospital, Bundang-gu, Seongnam-si, Gyeonggi-do 13620 Republic of Korea; 2 Department of Internal Medicine, Seoul National University College of Medicine, Seoul, 03080 Republic of Korea

**Keywords:** Cancer, Cell biology, Molecular biology

## Abstract

Cell division cycle 6 (*Cdc6*) plays key roles in regulating DNA replication, and activation and maintenance of cell cycle check points. In addition, Cdc6 exerts oncogenic properties via genomic instability associated with incomplete DNA replication. This study aimed to examine the effects of Cdc6 on pancreatic cancer (PC) cells. Our results showed that Cdc6 expression was higher in clinical PC specimens (based on analysis of the GEPIA database) and cell lines, and the high Cdc6 expression was associated with poorer survival in The Cancer Genome Atlas-PC cohort. In addition, Cdc6-depleted PC cells significantly inhibited cell proliferation and colony formation, delayed G_2_/M cell cycle progression, and increased expression of p-histone H3 and cyclin A2 levels. These observations could be explained by Cdc6 depletion leading to multipolar and split spindles via centrosome amplification and microtubule disorganization which eventually increases chromosome missegregation. Furthermore, Cdc6-depleted PC cells showed significantly increased apoptosis, which was consistent with increased caspase-9 and caspase-3 activation. Collectively, our results demonstrated that Cdc6-depleted PC cells are arrested in mitosis and eventually undergo cell death by induced multipolar spindles, centrosome aberrations, microtubule disorganization, and chromosome instability. In conclusion, Cdc6 may be a potential biomarker and therapeutic target for PC.

## Introduction

Pancreatic cancer (PC) is the leading cause of cancer-related death worldwide. Due to the lack of early cancer-related symptoms, the majority of patients are diagnosed at an advanced disease stage. Despite recent advances in surgical techniques, chemotherapy, and radiation therapy the five-year survival rate remains at just 8.7%^1,2^.

The checkpoint mechanism of the cell division cycle is tightly controlled in normal cells as it arrests cell cycle progress and allows time for DNA damage repair^3^. However, cancer cells are characterized by cell cycle dysregulation associated with increased DNA replication and cellular proliferation, despite the presence of DNA replication stress or DNA damage^4^. Cell division requires the precise coordination and execution of several events in the cell cycle, including centrosome duplication, DNA replication, mitotic spindle assembly, chromosome segregation, and cytokinesis. The proper coordination of these events ensures faithful chromosome segregation and avoids aneuploidy or polyploidy^5,6^. For example, centrosomes are the main microtubule organizing centers in the G_1_ and G_2_ phases of the cell cycle and play a critical role in mitotic spindle orientation and genome stability^7–9^. The presence of more than two centrosomes can cause multipolar distorted spindles and aberrant chromosome segregation, which induce cell cycle G_2_/M arrest and cell death due to mitotic failure^10^. Therefore, understanding and targeting the cancer cell cycle mechanism has potential therapeutic value for treating PC.

Cell division cycle 6 (*Cdc6*) is a key component of the pre-replication complex (pre-RC) that initiates DNA replication and plays important roles in the activation and maintenance of cell cycle checkpoint mechanisms. During early G_1_ phase, Cdc6 is also a component of pre-RCs assembled with the replication licensing factors (RLFs) ORC, Cdt1, and MCM2-7, which form origins of DNA replication and initiate DNA replication during S phase^11^. Cdc6 is also involved in checkpoint mechanisms that coordinate S phase in the cell cycle and mitotic entry. By coupling DNA replication and the cell cycle S-M phase checkpoint, Cdc6 ensures that the entire genome is replicated just once per cell division^12^. Interestingly, recent studies have shown that Cdc6 is also required for proper centrosome duplication; its interaction with Sas-6, which is regulated by Plk4, suppresses centrosome over-duplication^13^, while its depletion increases pericentriolar material (PCM) protein levels at the centrosome^14^. Many previous studies have indicated that abnormal Cdc6 expression plays an important role in brain tumors^15^, hepatocellular carcinoma^16^, breast cancer^17^, gastric cancer^18^, lung cancer^19^, ovarian cancer^20,21^, prostate cancer^22^, mitotic slippage, and drug resistance in bladder cancer and neuroblastoma^23,24^. Moreover, Cdc6 knockdown leads to increased DNA damage in Kras mutant cells^25^; however, the significance of Cdc6 in the progression of PC remains unknown.

In this study, we investigated whether Cdc6 expression was significantly associated with PC progression, and if it induced centrosome abnormalities, microtubule disorganization, and chromosome instability. We showed that Cdc6 may be a potential anticancer target and may help to understand the mechanism of PC progression.

## Results

### Expression of Cdc6 in PC patients and cell lines

**Figure 1:**
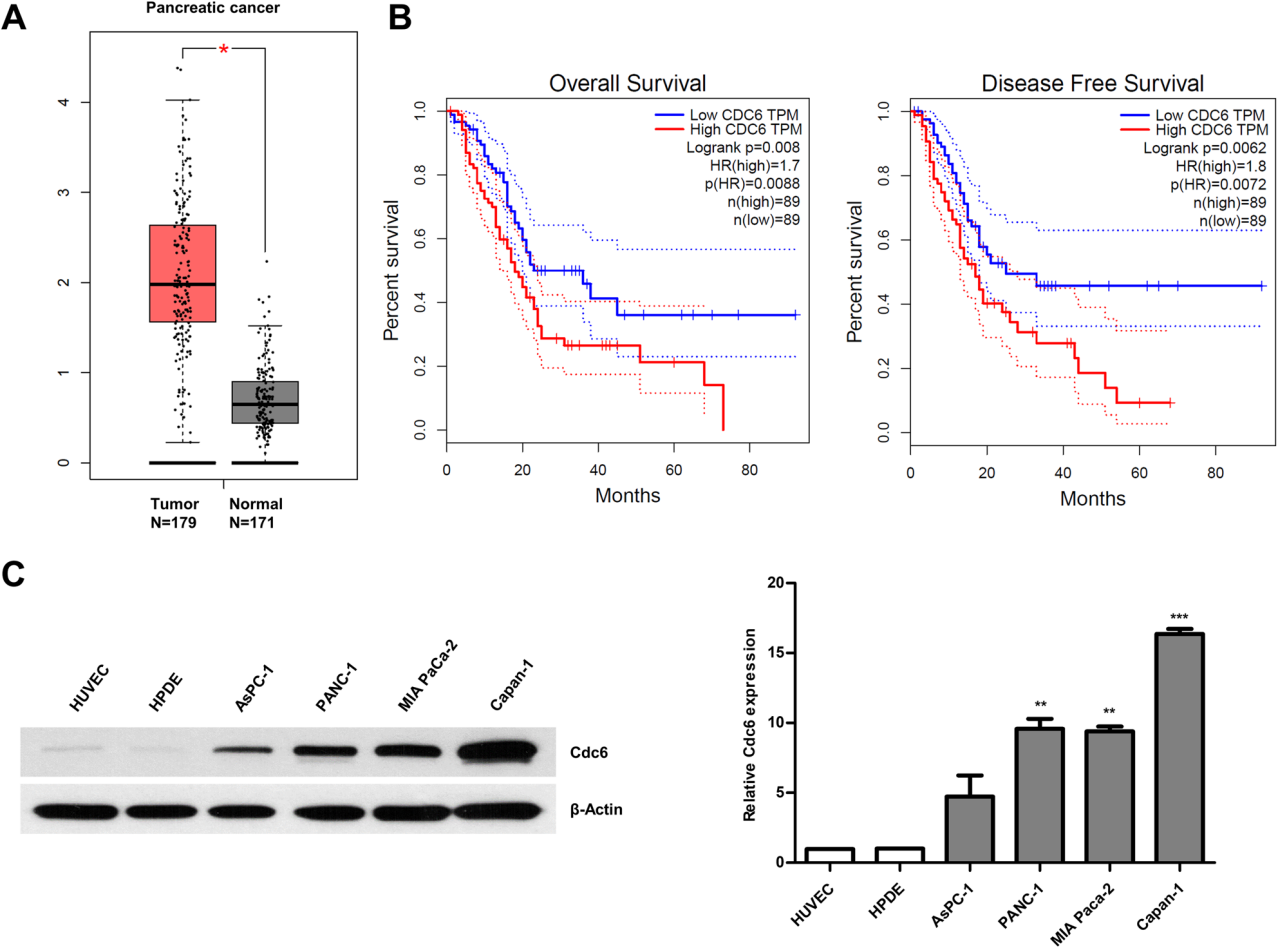
Cdc6 is upregulated in PC and correlates with clinical effects. (**A**) Cdc6 mRNA expression levels in PC tissues (n = 179) and normal tissues (n = 171) based on the TCGA and GTEx database. Each dot represents Cdc6 expression in one sample. (**B**) The OS and DFS of PC patients with low or high Cdc6 expression levels were analyzed using the Kaplan–Meier method and a log rank test in PC. Median values are indicated by full lines. *P < 0.005. TCGA, The Cancer Genome Atlas; TPM, transcripts per million; OS, overall survival; DSF, disease free survival; HR, hazard ratio. (**C**) Cdc6 expression was detected in PC cell lines (AsPC-1, PANC-1, MIA PaCa-2 and Capan-1 cells), HUVEC, and HPDE pancreatic cells by western blotting. The Cdc6/β-actin ratio was determined by densitometric analysis using ImageJ. Error bars represent standard deviations of the means of three biological replicates. Statistical analysis was performed using *t*-test. **p < 0.001, ***p < 0.0001.

To determine the clinical relevance of Cdc6 expression in PC, we analyzed mRNA expression of Cdc6 in clinical PC tissues from the publicly available Gene Expression Profiling Interactive Analysis (GEPIA) database. Our results showed that Cdc6 mRNA levels were significantly higher in PC tissues (n = 179) than normal tissues (n = 171) from The Cancer Genome Atlas (TCGA) and GTEx data (Fig. 1A). The overall survival (OS) and disease-free survival (DFS) data demonstrated that high Cdc6 expression contributed to significantly poorer survival in PC patients (Fig. 1B). Next, we analyzed the expression of Cdc6 in PC cell lines by western blot analysis, and observed that Cdc6 was up-regulated in various PC cell lines including AsPC-1, PANC-1, MIA PaCa-2, and Capan-1 cells compared to human umbilical vein endothelial cells (HUVEC) and normal human pancreatic duct epithelial (HPDE) cells (Fig. 1C). These results suggest that Cdc6 is aberrantly overexpressed in human PC cells and influences the survival of patients with PC.

### Cdc6 depletion inhibits cell proliferation, colony formation, and induces G_2_/M cell cycle arrest in PC cells

**Figure 2:**
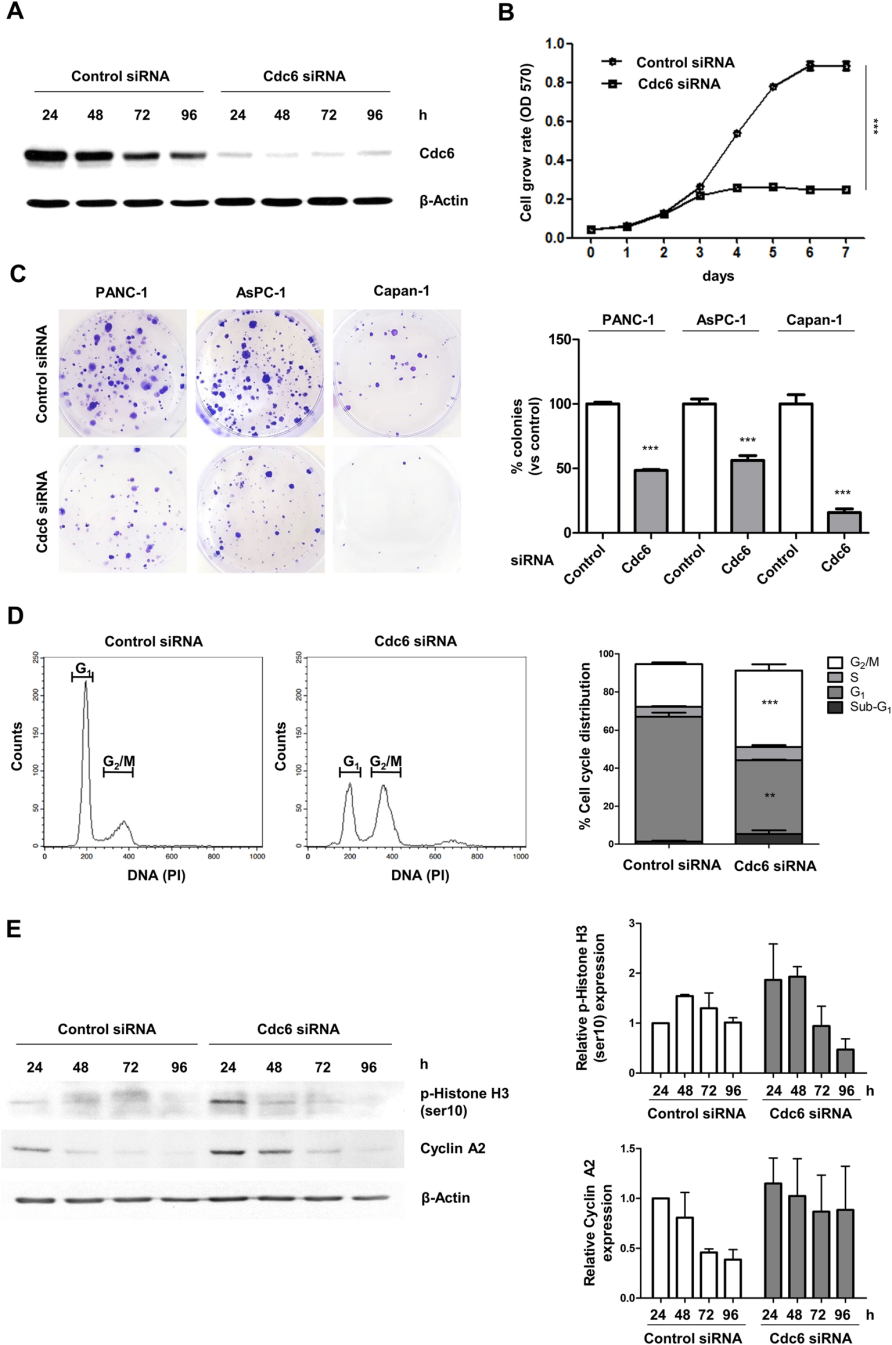
Cdc6 depletion suppresses PC cell proliferation and colony formation and induces G_2_/M cell cycle arrest. (**A**) PANC-1 cells were transfected with siRNA specific for Cdc6 or control. The level of Cdc6 proteins were confirmed by western blotting. Cells were harvested at the indicated time and lysates were immunoblotted with the indicated antibodies. (**B**) The effects of Cdc6 depletion on PANC-1 cell proliferation were determined using MTT assays. Cells were transfected with control and Cdc6 siRNA for 7 days. Error bars represent standard deviations of the means of three biological replicates. Statistical analysis was performed using two-way analysis of variance. ***p < 0.0001. (**C**) Colony formation assay was performed in PANC-1, AsPC-1 and Capan-1 cells with control siRNA and Cdc6 siRNA. The colonies were imaged and counted. Error bars represent standard deviations of the means of three biological replicates. Statistical analysis was performed using *t*-test. ***p < 0.0001. (**D**) Flow cytometric profiles of PANC-1 cells with control and Cdc6 siRNA for 72 h. Cells were harvested and analyzed to determine the percentage of cells in sub-G_1_, G_1,_ S, and G_2_/M phase. Relative quantitative analysis data from a representative of three experiments are shown. Statistical analysis was performed using *t*-test. **p < 0.001, ***p < 0.0001. (**E**) Western blot analysis of the cell cycle-related proteins p-histone H3 and cyclin A2 in PANC-1 cells. Cells were harvested at the indicated time and lysates were immunoblotted with the indicated antibodies. The p-histone H3/β-actin and cyclin A2/β-actin ratios were determined by densitometric analysis using ImageJ.

To evaluate the effects of Cdc6 knockdown on the survival and growth of PC cells, we employed colony formation assays and 3-(4,5**-**dimethylthiazol**-**2**-**yl)-2,5-diphenyltetrazolium bromide (MTT) assays. First, we transfected PANC-1 cells with three Cdc6 siRNAs (Cdc6_1, Cdc6_2, and Cdc6_3). Cdc6_1 siRNA than Cdc6_2 siRNA or Cdc6_3 siRNA resulted in significant inhibition of Cdc6 expression of PANC-1 cells (Supplementary Fig. 1A). Also, Cdc6_1 siRNA and Cdc6_3 siRNA resulted in significant inhibition of proliferation of PANC-1 cells (Supplementary Fig. 1B). Therefore, Cdc6_1 siRNA was used in subsequent experiments. Next, the results of western blot analysis showed that Cdc6 expression was effectively reduced in Cdc6 siRNA-transfected cells for up to 96 h (Fig. 2A and Supplementary Fig. 1C). PC cell proliferation was markedly reduced by Cdc6 depletion compared with the control (Fig. 2B), and Cdc6 knockdown significantly inhibited colony formation in PANC-1, AsPC-1, and Capan-1 cells (Fig. 2C). These results indicate that Cdc6 plays an important role in the proliferation of PC cells.

To further characterize the role of Cdc6 in the cell cycle, we analyzed the cell cycle distribution of Cdc6 siRNA-transfected PANC-1 and control-transfected cells by flow cytometry analysis with PI staining 72 h after transfection. We observed that Cdc6 depletion led to the accumulation of cells in G_2_/M phase (Fig. 2D), increased the expression of histone H3 phosphorylation (Ser10) (a mitotic marker), and maintained cyclin A2 expression (a cell cycle marker whose expression is known to increase in G_2_ phase) for longer than the control (Fig. 2E). These results indicate that Cdc6 depletion increases expression of the cell cycle-related proteins p-histone H3 and cyclin A2, and induces G_2_/M cell cycle arrest.

### Cdc6 depletion promotes apoptosis

**Figure 3:**
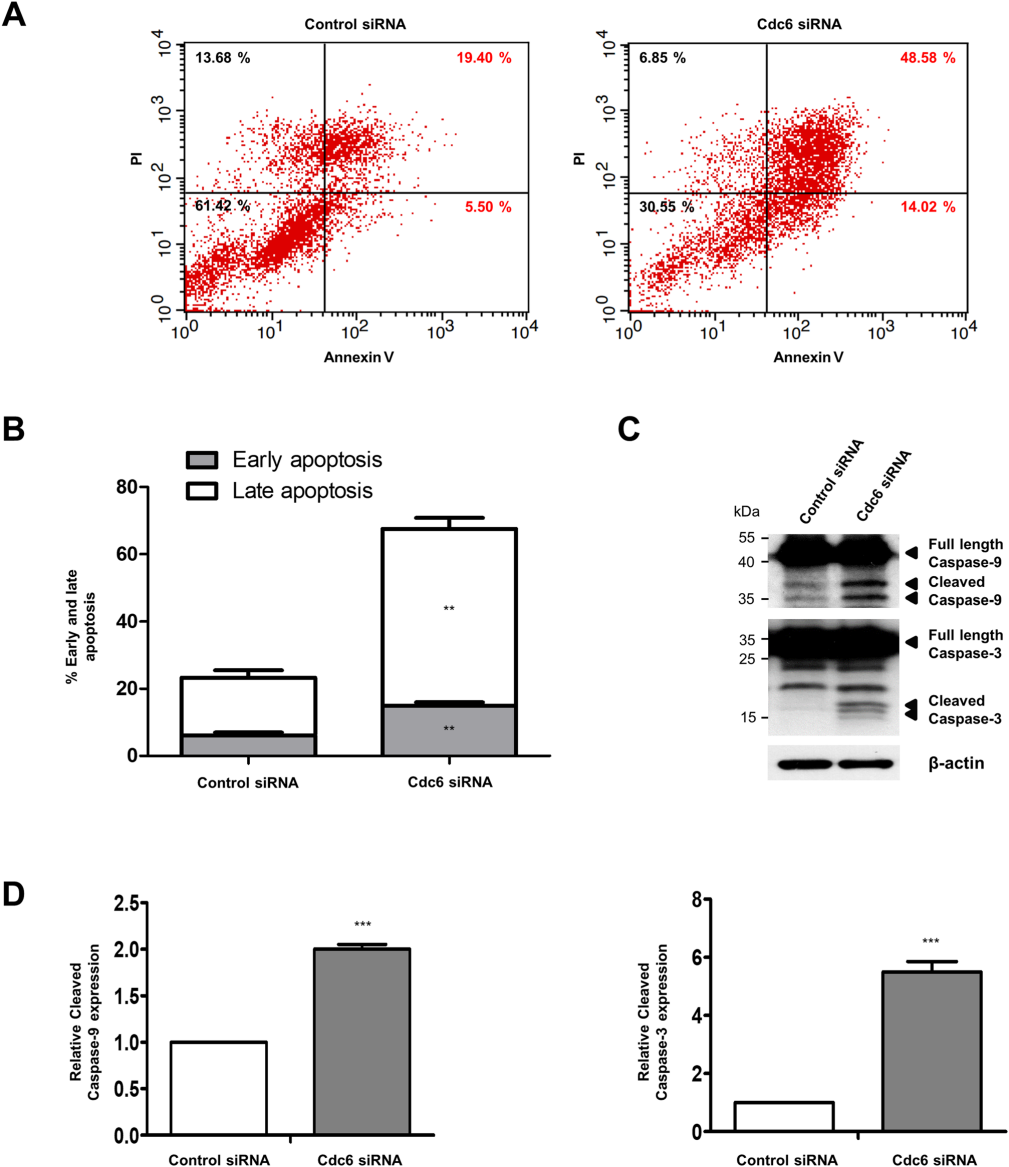
Cdc6 depletion induces apoptosis in PC cells. (**A**) Apoptotic cells were detected by Annexin V-FITC and PI staining using flow cytometry of PANC-1 cells with control or Cdc6 siRNA. Early apoptotic cells stained as Annexin V-positive and PI-negative (lower right), and late apoptotic cells stained as Annexin V-positive and PI-positive (upper right). (**B**) The percentage of cells including early apoptotic cells and late apoptotic cells. Relative quantitative analysis data from a representative of three experiments are shown. Statistical analysis was performed using *t*-test. **p < 0.001. (**C**) Western blot analysis was performed to determine the expression of the apoptosis-related proteins caspase-9 and caspase-3. Cells were harvested at the indicated time and lysates were immunoblotted with the indicated antibodies. (**D**) The cleaved caspase-9/β-actin and cleaved caspase-3/β-actin ratios were determined by densitometric analysis using ImageJ. Error bars represent standard deviations of the means of three biological replicates. Statistical analysis was performed using *t*-test. ***P < 0.0001.

To explore the effects of Cdc6 on the apoptosis of PC cells, we performed staining analysis using annexin V-fluorescein isothiocyanate (FITC), which recognizes phospholipid phosphatidylserine on the outer membrane of apoptotic cells, and propidium iodide (PI), a marker of cell membrane permeability^26^. Annexin V-FITC(+)/PI(−) staining indicated the early apoptotic cells, while annexin V-FITC(+)/PI(+) staining revealed the late apoptotic cells. The number of apoptotic cells significantly increased when transfected with Cdc6 siRNA compared with the control. As a result, the percentages of early and late apoptotic cells were significantly increased to 14.99 ± 1.72% and 52.6 ± 5.6%, respectively, after Cdc6 siRNA transfection compared with 6.1 ± 1.47% and 17.23 ± 3.08%, respectively, in the control-transfected cells (Fig. 3A,B). Caspase-9 and caspase-3 have distinct roles in the intrinsic apoptotic pathways. First, caspase-9 is activated by mitochondria-released cytochrome c, and then caspase-3 is activated^27^. The upregulation of apoptosis in Cdc6-depleted cells was confirmed by western blotting; caspase-9 and caspase-3 protein expression were observed 48 h after transfection and the cleaved form was highly expressed in Cdc6-depleted cells (Fig. 3C,D). Thus, Cdc6 depletion appears to induce cell cycle arrest in PC cells, followed by apoptotic cell death.

### Cdc6 depletion induces centrosome and spindle abnormalities in PC cells

**Figure 4:**
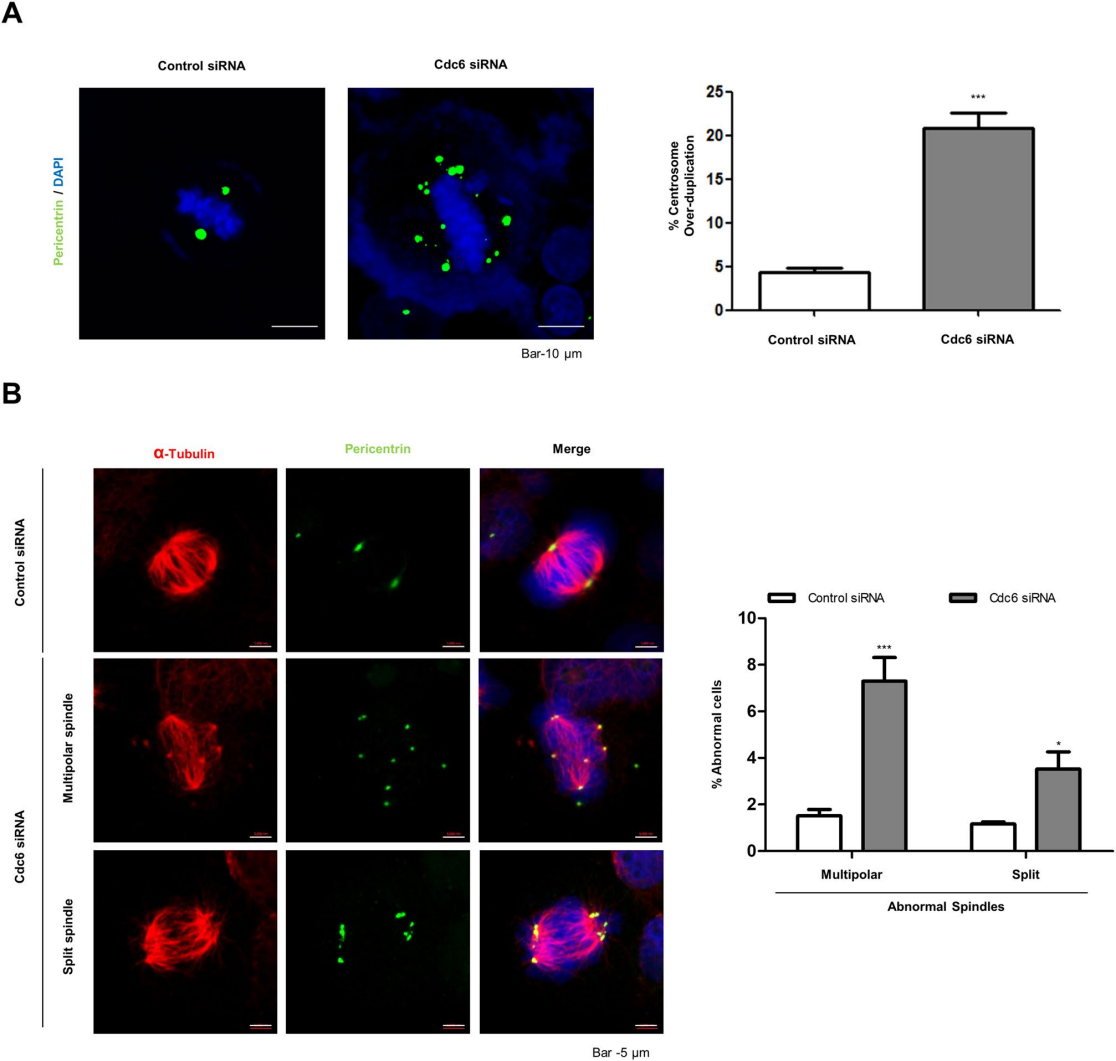
Cdc6 depletion causes centrosome abnormalities in PC cells. (**A**) Immunofluorescence staining of PANC-1 cells transfected with control or Cdc6 siRNA with pericentrin (green) antibodies and DAPI (blue). Graph represents the percentage of cells with centrosome over-duplication and chromosome abnormalities. Scale bar, 10 µm. Relative quantitative analysis data from a representative of three experiments are shown. Statistical analysis was performed using *t*-test. ***p < 0.0001. (**B**) Immunofluorescence staining of PANC-1 cells transfected with control or Cdc6 siRNA with α-tubulin (red), pericentrin (green) antibodies, and DAPI (blue). The graph represents the percentage of cells with abnormal spindles into multipolar and split spindles. Scale bar, 5 µm. Data are representative of three different fields in each independent experiment. Statistical analysis was performed using *t*-test. *P < 0.005, ***p < 0.0001.

According to previous research, Cdc6 localizes to the centrosome during the S and G_2_ phases of the cell cycle, while a lack of Cdc6 has been shown to cause centrosome over-duplication in U2OS cells^13,14^. Hence, we examined whether Cdc6 depletion induced centrosome over-duplication by transfecting PANC-1 cells with Cdc6 siRNA and staining them with pericentrin (centrosome marker). Immunofluorescence confocal microscopy revealed that two normal centrosomes were found in control cells, whereas two or more abnormal centrosomes were found in Cdc6 siRNA-transfected cells (Fig. 4A). In addition, to investigate the effect of cell death on over-duplication with the centrosome, immunofluorescence analysis was performed using cleaved caspase-3 (apoptosis marker) and pericentrin antibodies. As a result, we could observe an increase in the expression of the cleaved caspase-3 antibody in cells with increased centrosomes (Supplemental Fig. 2A). Since centrosomes play an important role in microtubule organization, we analyzed severe spindle abnormalities in mitotic cells following Cdc6 depletion in PC cells. We divided spindle abnormalities into multipolar and split spindles. Multipolar spindles have at least three half-spindles and/or astral structures, while split spindles have two pair of centrioles, but at least one pair of centrioles separated by ≥ 2 μm. We measured a 7.3% incidence of multipolar spindles and a 3.5% incidence of split spindles in Cdc6-deficient mitotic cells. Overall, we observed a 10.8% occurrence of all spindle abnormalities, which was a four times greater incidence compared to the control (Fig. 4B). These data suggest that Cdc6 depletion interferes with normal centrosome division, leading to spindle abnormalities.

### Cdc6 depletion leads to chromosome missegregation

**Figure 5:**
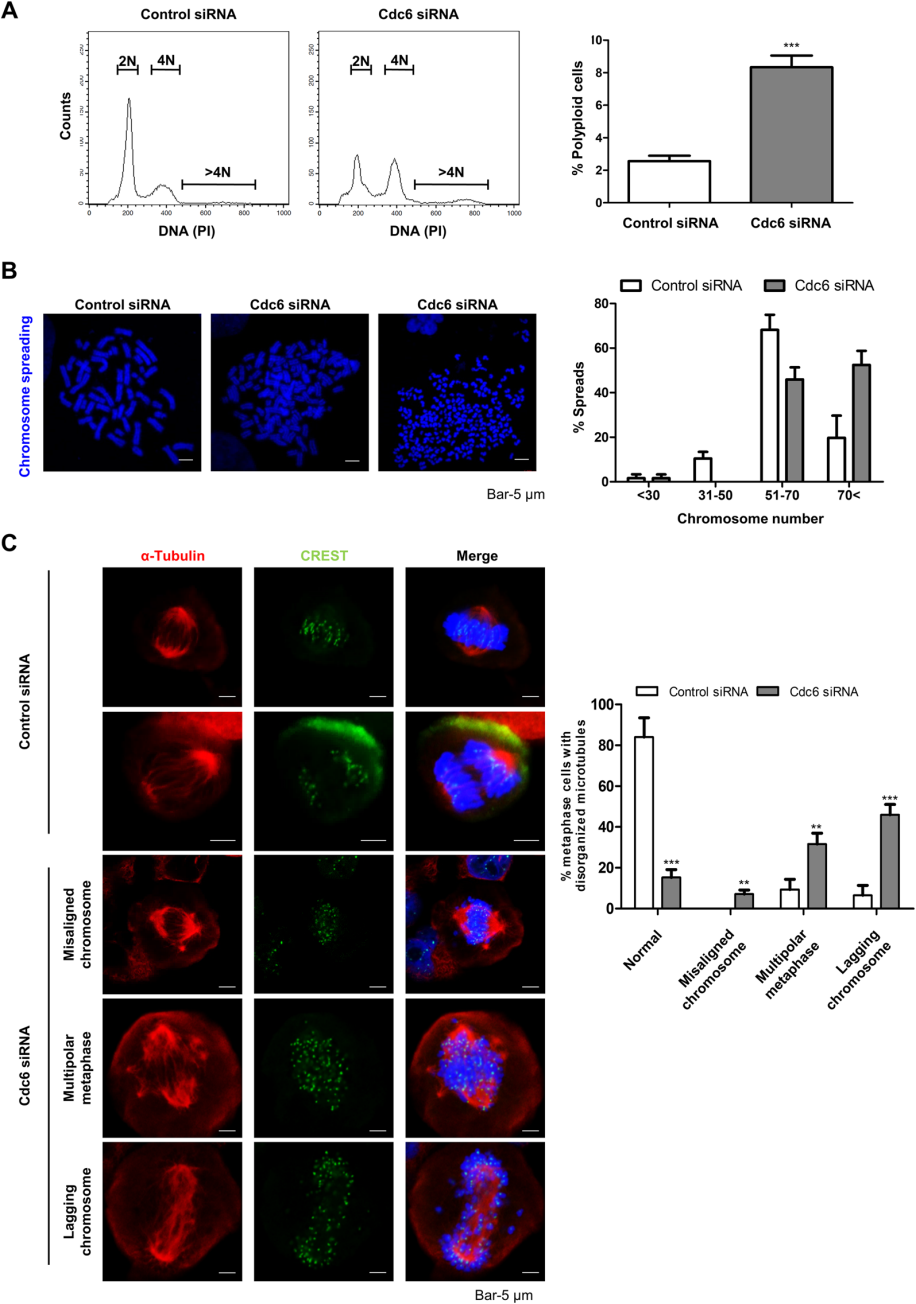
Cdc6 depletion induces chromosome instability. (**A**) Flow cytometry of polyploidy cells (> 4 N DNA content) with control or Cdc6 siRNA in PANC-1 cells for 96 h. Graph represents the percentage of polyploidy cells. Statistical analysis was performed using *t*-test. ***p < 0.0001. (**B**) Aneuploidy cells were represented by metaphase chromosome spread analysis with control or Cdc6 siRNA in PANC-1 cells for 72 h. Spread chromosomes were observed by staining with DAPI and chromosome number divided aneuploidy into < 30, 31–50, 51–70, and > 70 chromosome numbers. Scale bar, 5 µm. (**C**) Immunofluorescence staining of PANC-1 cells transfected with control or Cdc6 siRNA with α-tubulin (red), CREST (green) antibodies, and DAPI (blue). The percentage of cells with misaligned chromosome, multipolar metaphase, and lagging chromosome was measured. Scale bar, 5 µm. Data are representative of three different fields were analyzed each independent experiment. Statistical analysis was performed using *t*-test. **p < 0.001, ***p < 0.0001.

We observed that Cdc6-deficiency in cells lead to an increase in DNA content 4 N or even greater suggesting polyploidy cells (Fig. 2D). In order to analyze the effect of Cdc6 on the formation of polyploidy cells, control siRNA- and Cdc6 siRNA-transfected PC cells were analyzed by flow cytometry after 96 h. As expected, Cdc6-deficient PC cells induced polyploidy (Fig. 5A). Next, using a metaphase chromosome spreading analysis to measure the number of chromosomes, we determined that aneuploidy (more than 70 chromosomes) occurred in 52.46% of Cdc6-deficient PC cells. On the other hand, in control siRNA-transfected PC cells, aneuploidy was observed in only 19.72% of the cells (Fig. 5B). Moreover, we examined whether defects in chromosome segregation in Cdc6-depleted PC cells might be due to abnormalities in the attachment between microtubules and the kinetochore by immunostaining with CREST and α-tubulin antibodies. In the control siRNA PC cells, α-tubulin was normally attached to CREST. However, in most of the Cdc6-depleted PC cells, α-tubulin extended in several directions rather than toward both ends and was not attached to CREST, indicating that Cdc6 depletion caused chromosome segregation error (Fig. 5C). As a result of analyzing the disorganized microtubules of cells dissociated into misaligned chromosomes, multipolar metaphase, and lagging chromosomes in the total metaphase cells, the number of misaligned chromosomes increased 5.6-fold in Cdc6-deficient PC cells compared to that in control siRNA cells. Multipolar metaphase was observed to increase more than 3.7-fold and lagging chromosome increased more than 7.1-fold in Cdc6-deficient PC cells compared to that in control siRNA cells (Fig. 5C). Thus, these data demonstrate that Cdc6 depletion causes multipolar spindles and leads to chromosome instability.

## Discussion

Cdc6 has been reported as a potential therapeutic target in many cancer types, and studies on its role in various cancers have been conducted^21,28,29^. However, the role of Cdc6 in PC has not been reported, and the mechanisms involved are unclear. In this study, we showed that Cdc6 was upregulated in PC cell lines and high expression of Cdc6 is related to clinical outcomes. Cdc6 depletion not only inhibited cell proliferation but also resulted in G_2_/M cell cycle arrest with upregulation of p-histone H3 and cyclin A2 in PC cells. Similar to our results, Cdc6 downregulation inhibited cell proliferation, DNA synthesis, epithelial-mesenchymal transition (EMT), migration, and invasion in human colorectal cancer^30^. Also, Cdc6 was reported to be highly expressed in osteosarcoma patients and osteosarcoma cell lines, and proliferation was inhibited in MG63 cells due to the deficiency of Cdc6^29^. However, in osteosarcoma cells, G_1_ cell cycle arrest was observed, and the expression of cyclin D1 and cyclin A2 was decreased due to Cdc6 deficiency. This difference in results indicates that the inhibition of cell proliferation occurs due to the deficiency of Cdc6 in PC cells and osteosarcoma but the mechanisms underlying these effects are different. Moreover, we found that Cdc6 depletion induces a series of abnormal events, including centrosome over-duplication, multipolar spindle formation, and chromosome instability. These results are similar those reported in a previous study that Cdc6 and Plk4 are co-localized at the centrosome and have an important role in efficient centrosome duplication during the cell cycle^13^. Cdc6 depletion promoted apoptotic cell death with increased activation of caspase-3 and caspase-9. These data suggest that Cdc6 can play an important role in the proliferation, cell cycle, and death of PC cells. To the best of our knowledge, this is the first report show that Cdc6 contributes to PC cell progression.

Centrosome duplication must be tightly regulated to avoid multipolar spindle assembly and genome instability during each cell cycle^31^. The duplicated centrosome is separated during the previous stages of mitosis and moves to the opposite pole, functioning as a mitotic spindle pole for chromosome separation. Later in mitosis, they are separated into individual daughter cells using chromosome segregation. If centrosome duplication and function are not controlled, they may lead to multipolar spindle formation, aneuploidy, asymmetric cell division and cell polarity destruction^32^. According to a recent study, Cdc6 is located at the centrosome during the S and G_2_ phases of the cell cycle^33^. The function of centrosomal Cdc6 is to inhibit recruitment of PCM proteins such as γ-tubulin, pericentrin, CDK5RAP2, and Cep192 to the centrosome. Other studies have shown that Cdc6 controls centrosome duplication regardless of the mobilization of the PCM protein to the centrosome^34^. In addition, the interaction of Sas-6 and Cdc6, which is regulated by Plk4 phosphorylation of Cdc6, inhibits over-duplication^13^. Consistently, we observed that the number of centrosomes increased due to Cdc6 deficiency, thereby increasing cell aneuploidy.

In conclusion, our study shows that Cdc6 depletion in PC cells inhibit cell proliferation, induces G_2_/M arrest, increases centrosome over-duplication leading to multipolar spindles and microtubule disorganization, chromosome instability, and ultimately, encourages cell death. Thus, Cdc6 may serve as a promising therapeutic target for treating PC.

## Materials and methods

### Database analysis

Expression levels of Cdc6 in PC and normal pancreases were compared by Gene Expression Profiling Interactive Analysis (GEPIA) (https://gepia.cancer-pku.cn/)^35^ of 179 PC tissues and 171 normal tissues from Cancer Genome Atlas (TCGA)^36^ and Genotype-Tissue Expression (GTEx) normal samples^37,38^. Transcripts per million (TPM) were determined, and gene expression levels were presented using a log_2_ (TPM + 1) scale. The cutoff values were 1 for |Log_2_FC| and 0.01 for the *P* value, and the OS of patients with PC was also assayed.

### Cell culture

The human PC cell lines AsPC-1, PANC-1, MIA Paca-2, and Capan-1 were obtained from the American Type Culture Collection (ATCC, Manassas, VA, USA) and KCLB (Korea Cell Line Bank, Seoul, South Korea). Human Umbilical Vein Endothelial Cells (HUVEC) are obtained from Lonza (Lonza, Basel, Switzerland). The non-cancerous immortalized human pancreatic ductal epithelial (HPDE) cell line was obtained from Joo Kyung Park (MD, Samsung Medical Center, Seoul, South Korea). MIA Paca-2 and PANC-1 cells were cultured in Dulbecco’s Modified Eagle Medium, while AsPC-1, and Capan-1 cells were cultured in RPMI-1640 (Gibco-Invitrogen, Carlsbad, CA, USA) supplemented with 10% fetal bovine serum with 1% penicillin and streptomycin. Cells were maintained in a humidified 5% CO_2_ atmosphere at 37 °C. HPDE cells were maintained in keratinocyte serum-free medium supplemented with epidermal growth factor and bovine pituitary extract (Gibco-Invitrogen).

### RNA interference

Cdc6-specific siRNA with the following sequence: Cdc6_1: 5ʹ-AAC UUC CCA CCU UAU ACC AGA-3ʹ^39^, Cdc6_2: 5ʹ-AAG AAU CUG CAU GUG UGA GAC-3ʹ^40^ and Cdc6_3: 5ʹ-CCA AGA AGG AGC ACA AGA U-3ʹ^41^ were synthesized by GenePharma (Shanghai, China). Cells were transfected with the siRNA using Lipofectamine RNAiMAX transfection reagents according to the manufacturer’s instructions (Invitrogen, Carlsbad, CA, USA).

### Cell proliferation and colony formation assays

PANC-1 cells were seeded in 12-well plates at a density of 2 × 10^4^ cells per well. After siRNA transfection, cell proliferation was monitored every 24 h for 7 days using MTT (Sigma-Aldrich, Saint Louis, MO, USA) assay. Briefly, 50 μL of prepared MTT solution was added to each well at the desired time point and incubated at 37 °C for 4 h. The media was carefully removed and the cells were solubilized in 500 μL of dimethyl sulfoxide (DMSO). Plates were read spectrophotometrically at a wavelength of 570 nm. For the colony formation assay, 1 × 10^3^ PANC-1 cells were seeded in a six-well plate and transfected with siRNA. After 2 weeks, the colonies were fixed with methanol, stained with 0.1% crystal violet (Sigma-Aldrich), and counted.

### Flow cytometric analysis of the cell cycle and apoptosis

To analyze the cell cycle, cells were collected, fixed with 80% cold ethanol, and maintained at 4 °C overnight. The cells were then treated with 50 µg/mL RNAse A, stained with 50 µg/mL PI, and analyzed by flow cytometry (BD Biosciences, San Jose, CA, USA). To assess apoptosis, the cells were double stained with an FITC Annexin V apoptosis detection kit (BD Biosciences) and analyzed according to the manufacturer’s instructions.

### Western blotting

Cells were lysed with radioimmunoprecipitation assay (RIPA) buffer (Cell Signaling Technology Inc., Danvers, MA, USA), a protease inhibitor cocktail (Sigma-Aldrich), and phenylmethylsulfonyl fluoride (PMSF, Cell Signaling Technology). Protein concentration was measured using the bicinchoninic acid (BCA) protein assay reagent (Pierce-Thermo scientific, Rockford, IL, USA). Equal amounts of protein from each cell lysate were separated on sodium dodecyl sulfate (SDS) polyacrylamide gels, transferred onto nitrocellulose (NE) membranes, and reacted with antibodies against p-histone H3 ser10 (Thermo Fisher Scientific, Waltham, MA, USA), cyclin A2 (Cell Signaling Technology), caspase-3 (Cell Signaling Technology), or caspase-9 (Cell Signaling Technology). The membranes were then washed with TBST (Tris-buffered saline, 0.1% Tween 20), incubated with HRP-conjugated anti-mouse IgG (The Jackson Laboratory, Bar Harbor, ME, USA) or anti-rabbit IgG (Cell Signaling Technology) secondary antibodies, and the target proteins were detected with ECL western blotting detection reagents (Amersham-GE Healthcare Life Sciences, Malborough, MA, USA). Total protein loading amounts and intensity were quantified using β-actin (Cell Signaling Technology) as the loading control.

### Immunofluorescence microscopy

PANC-1 cells were cultured in a Lab-Tek chamber slide (Nalge Nunc International, Rochester, NY, USA) at a density of 20,000 cells/well. After 48 h, cells were fixed with 4% paraformaldehyde in phosphate buffered saline (PBS) for 15 min. After being permeabilized with 0.5% Triton X-100 in PBS for 10 min, the cells were blocked with 1% BSA in PBS and incubated with primary antibodies overnight at 4 °C. Primary antibodies used in these studies were anti-pericentrin (Abcam, Cambridge, UK), anti-α-tubulin (Abcam), human anti-CREST (Immuno Vision Inc., Springdale, AR, USA) and anti-cleaved caspase-3 (1:400, Cell Signaling Technology). The cells were then washed three times with PBS, and incubated with the indicated secondary antibody for 2 h at 25 °C. Secondary antibodies were goat Alexa Fluor 568 (Invitrogen), goat Alexa Fluor 488 (Abcam), and goat anti-Human IgG-FITC (Invitrogen). Nuclei were counterstained with DAPI and mounted with ProLong Gold Antifade (Invitrogen). Images were captured using a ZEISS LSM 710 confocal microscope and processed using ZEN software (ZEISS International, Oberkochen, DE).

### Chromosome spreading assay

Cells were treated with colcemid (0.1 µg/mL) for 4 h and then harvested. After treatment with 0.075 M KCl and incubation at 37 °C, the cells were fixed with a dropwise application of a freshly-prepared methanol/acetic acid (3:1) solution and placed on glass slides. Slides were dried at room temperature, stained with DAPI (100 ng/mL), and mounted with ProLong Gold Antifade (Invitrogen). Images were captured using a ZEISS LSM 710 confocal microscope and processed using ZEN software (ZEISS International, Oberkochen, DE).

### Statistical analysis

All data represent average values obtained in form three independent experiments. Results were presented as means ± standard errors of means (SEMs). Statistical analyses were performed using GraphPad Prism software (version 5.0, GraphPad Software Inc., San Diego, CA, USA). Statistical comparisons were made using two-tailed unpaired *t*-tests and two-way analysis of variance (ANOVA). Results were considered significant when the* p*-value was *p < 0.005, **p < 0.001 or ***p < 0.0001.

## Supplementary information

Supplementary Figures.

## References

[1] Kamisawa T, Wood LD, Itoi T, Takaori K (2016). Pancreatic cancer. Lancet (London, England).

[2] N, H. *et al. SEER Cancer Statistics Review.* (Accessed 27 February 2019); https://seer.cancer.gov/csr/1975_2014/.

[3] Curtin NJ (2012). DNA repair dysregulation from cancer driver to therapeutic target. Nat. Rev. Cancer.

[4] Eastman A (2004). Cell cycle checkpoints and their impact on anticancer therapeutic strategies. J. Cell. Biochem..

[5] Rhind N, Russell P (2012). Signaling pathways that regulate cell division. Cold Spring Harbor Perspect. Biol..

[6] Negrini S, Gorgoulis VG, Halazonetis TD (2010). Genomic instability–an evolving hallmark of cancer. Nat. Rev. Mol. Cell Biol..

[7] Bornens M (2012). The centrosome in cells and organisms. Science (New York N.Y.).

[8] Gergely F, Basto R (2008). Multiple centrosomes: together they stand, divided they fall. Genes Dev..

[9] Godinho SA, Kwon M, Pellman D (2009). Centrosomes and cancer: how cancer cells divide with too many centrosomes. Cancer Metastasis Rev..

[10] Kramer A, Neben K, Ho AD (2002). Centrosome replication, genomic instability and cancer. Leukemia.

[11] Borlado LR, Mendez J (2008). CDC6: from DNA replication to cell cycle checkpoints and oncogenesis. Carcinogenesis.

[12] Clay-Farrace L, Pelizon C, Santamaria D, Pines J, Laskey RA (2003). Human replication protein Cdc6 prevents mitosis through a checkpoint mechanism that implicates Chk1. EMBO J..

[13] Xu X (2017). DNA replication licensing factor Cdc6 and Plk4 kinase antagonistically regulate centrosome duplication via Sas-6. Nat. Commun..

[14] Lee I (2017). The DNA replication protein Cdc6 inhibits the microtubule-organizing activity of the centrosome. J. Biol. Chem..

[15] Ohta S (2001). Cdc6 expression as a marker of proliferative activity in brain tumors. Oncol. Rep..

[16] Xiong XD (2008). A novel functional polymorphism in the Cdc6 promoter is associated with the risk for hepatocellular carcinoma. Mutat. Res..

[17] Mahadevappa R (2017). The prognostic significance of Cdc6 and Cdt1 in breast cancer. Sci. Rep..

[18] Zhao B, Zhang J, Chen X, Xu H, Huang B (2019). Mir-26b inhibits growth and resistance to paclitaxel chemotherapy by silencing the CDC6 gene in gastric cancer. Arch. Med. Sci. AMS.

[19] Zhang X (2014). MicroRNA-26a/b regulate DNA replication licensing, tumorigenesis, and prognosis by targeting CDC6 in lung cancer. Mol. Cancer Res. MCR.

[20] Deng Y (2016). High expression of CDC6 is associated with accelerated cell proliferation and poor prognosis of epithelial ovarian cancer. Pathol. Res. Pract..

[21] Sun TY, Xie HJ, He H, Li Z, Kong LF (2016). miR-26a inhibits the proliferation of ovarian cancer cells via regulating CDC6 expression. Am. J. Transl. Res..

[22] Liu Y, Gong Z, Sun L, Li X (1839). FOXM1 and androgen receptor co-regulate CDC6 gene transcription and DNA replication in prostate cancer cells. Biochem. Biophys. Acta.

[23] Chen S (2016). Cdc6 contributes to cisplatin-resistance by activation of ATR-Chk1 pathway in bladder cancer cells. Oncotarget.

[24] Feng L (2008). Cdc6 knockdown inhibits human neuroblastoma cell proliferation. Mol. Cell. Biochem..

[25] Steckel M (2012). Determination of synthetic lethal interactions in KRAS oncogene-dependent cancer cells reveals novel therapeutic targeting strategies. Cell Res..

[26] Rieger AM, Nelson KL, Konowalchuk JD, Barreda DR (2011). Modified annexin V/propidium iodide apoptosis assay for accurate assessment of cell death. J. Vis. Exp..

[27] Brentnall M, Rodriguez-Menocal L, De Guevara RL, Cepero E, Boise LH (2013). Caspase-9, caspase-3 and caspase-7 have distinct roles during intrinsic apoptosis. BMC Cell Biol..

[28] Hu Y (2019). Potential prognostic and diagnostic values of CDC6, CDC45, ORC6 and SNHG7 in colorectal cancer. OncoTargets Ther..

[29] Jiang W (2019). Downregulation of Cdc6 inhibits tumorigenesis of osteosarcoma in vivo and in vitro. Biomed. Pharmacother. Biomed. Pharmacother..

[30] Cai J (2019). The RNA-binding protein HuR confers oxaliplatin resistance of colorectal cancer by upregulating CDC6. Mol. Cancer Ther..

[31] Sluder G, Nordberg JJ (2004). The good, the bad and the ugly: the practical consequences of centrosome amplification. Curr. Opin. Cell Biol..

[32] Vitre BD, Cleveland DW (2012). Centrosomes, chromosome instability (CIN) and aneuploidy. Curr. Opin. Cell Biol..

[33] Kim GS, Kang J, Bang SW, Hwang DS (2015). Cdc6 localizes to S- and G2-phase centrosomes in a cell cycle-dependent manner. Biochem. Biophys. Res. Commun..

[34] Kim GS, Lee I, Kim JH, Hwang DS (2017). The replication protein Cdc6 suppresses centrosome over-duplication in a manner independent of Its ATPase activity. Mol. Cells.

[35] Tang Z (2017). GEPIA: a web server for cancer and normal gene expression profiling and interactive analyses. Nucl. Acids Res..

[36] Weinstein JN (2013). The Cancer Genome Atlas Pan-Cancer analysis project. Nat. Genet..

[37] The Genotype-Tissue Expression (GTEx) project. *Nat. Genet.* **45**, 580–585. 10.1038/ng.2653 (2013). 10.1038/ng.2653 PMC4010069 23715323

[38] Human genomics (2015). The Genotype-Tissue Expression (GTEx) pilot analysis: multitissue gene regulation in humans. Science (New York, N.Y.).

[39] Habedanck R, Stierhof YD, Wilkinson CJ, Nigg EA (2005). The Polo kinase Plk4 functions in centriole duplication. Nat. Cell Biol..

[40] Melixetian M (2004). Loss of Geminin induces rereplication in the presence of functional p53. J. Cell Biol..

[41] He Y (2016). Cell division cycle 6 promotes mitotic slippage and contributes to drug resistance in paclitaxel-treated cancer cells. PLoS ONE.

