# Supplementary material for: Cdc6 disruption leads to centrosome abnormalities and chromosome instability in pancreatic cancer cells
Source: Sci Rep. 2020 Oct 5;10:16518. doi: 10.1038/s41598-020-73474-6 (PMC7536414; doi:10.1038/s41598-020-73474-6)
Supplement: Supplementary file 1 — Supplementary Figures. [file 41598_2020_73474_MOESM1_ESM.pdf]

Cdc6 disruption leads to centrosome abnormalities and chromosome instability in pancreatic cancer cells.

Yuna Youn[1], Jong-chan Lee[1,2], Jaihwan Kim[1], Jae Hyeong Kim[1*], Jin-Hyeok Hwang[1,2*]

[1] Department of Internal Medicine, Seoul National University Bundang Hospital, Seongnam-si, Gyeonggi-do, 13620, Republic of Korea

[2] Department of Internal Medicine, Seoul National University College of Medicine, Seoul, 03080, Republic of Korea

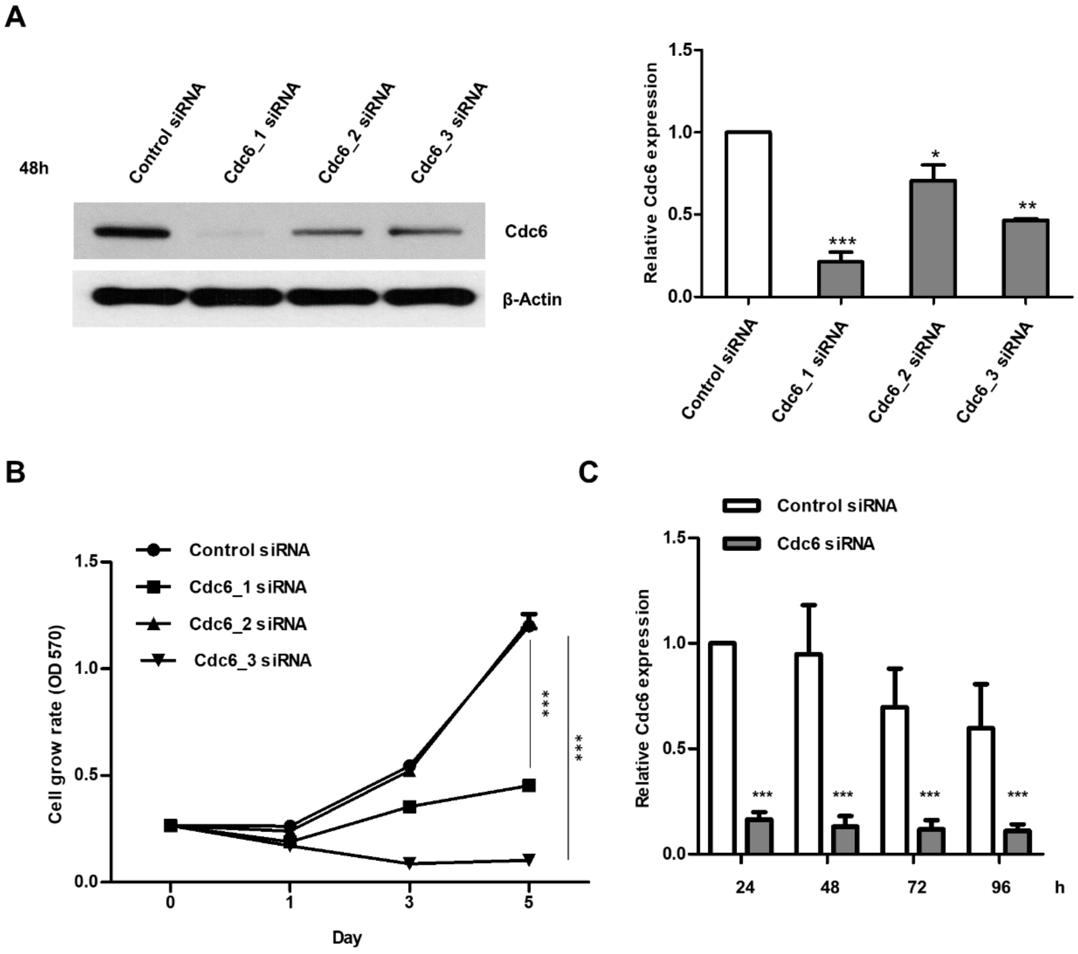

Supplementary Figure 1. Cdc6 depletion suppresses protein expression and PC cell proliferation.

(A) PANC-1 cells were transfected with siRNA specific for control, Cdc6_1, Cdc6_2 or Cdc6_3 siRNA. The level of Cdc6 proteins were confirmed by western blotting. Cells were harvested at 48 h later and lysates were immunoblotted with the indicated antibodies. The Cdc6/β-actin ratio was determined by densitometric analysis using ImageJ. Error bars represent standard deviations of the means of three biological replicates. Statistical analysis was performed using *t*-test. *$P < 0.005$, **$P < 0.001$, and ***$P < 0.0001$. **(B)** The effects of

Cdc6 depletion on PANC-1 cell proliferation were determined using MTT assays. Cells were transfected with control, Cdc6_1, Cdc6_2 or Cdc6_3 siRNA for 5 days. Error bars represent standard deviations of the means of three biological replicates. Statistical analysis was performed using two-way analysis of variance. ***$p < 0.0001$. **(C)** The Cdc6/β-actin ratio was determined by densitometric analysis using ImageJ. Error bars represent standard deviations of the means of three biological replicates. Statistical analysis was performed using *t*-test. ***$p < 0.0001$.

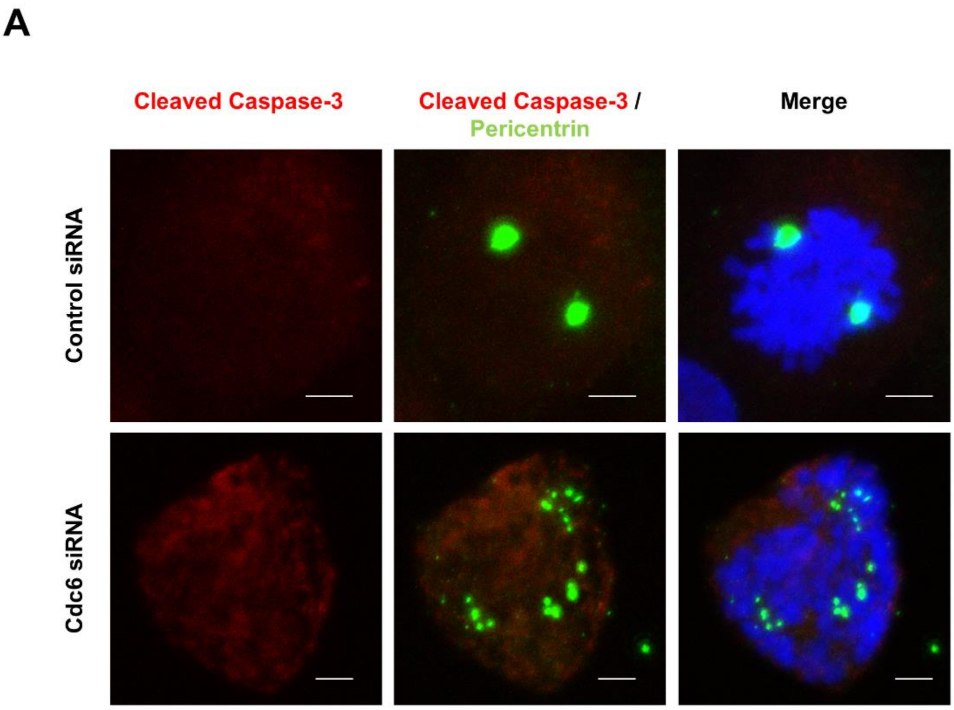

Supplementary Figure 2. Cdc6 depletion causes apoptosis on centrosome over-duplication.

(A) Immunofluorescence staining of PANC-1 cells transfected with control siRNA or Cdc6 siRNA with cleaved caspase-3 (red), pericentrin (green) antibodies, and DAPI (blue). Scale bar, 5 µm.

Figure 1. C

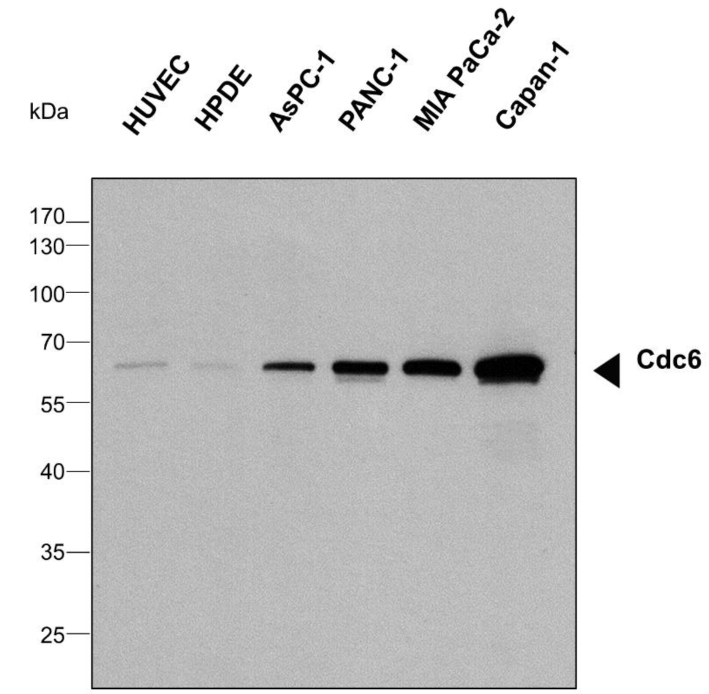

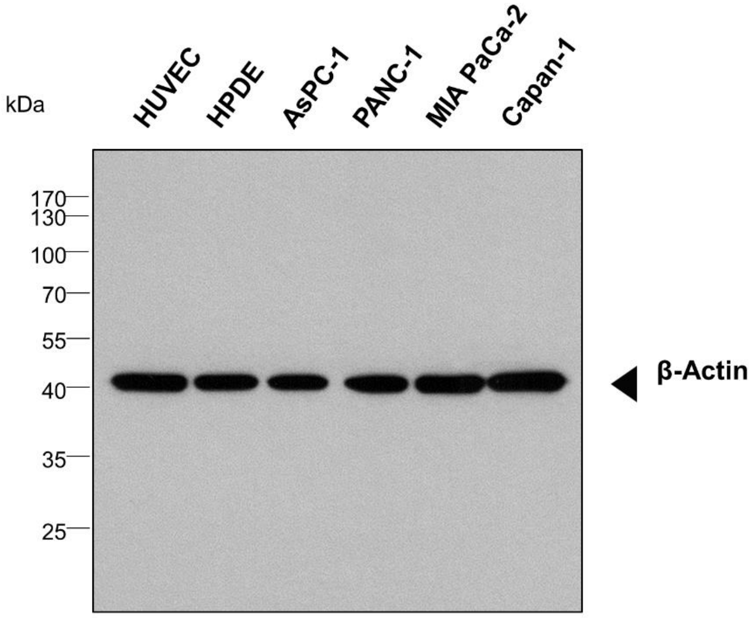

Supplementary Figure 3. Full-length immunoblots.

Figure 2. A

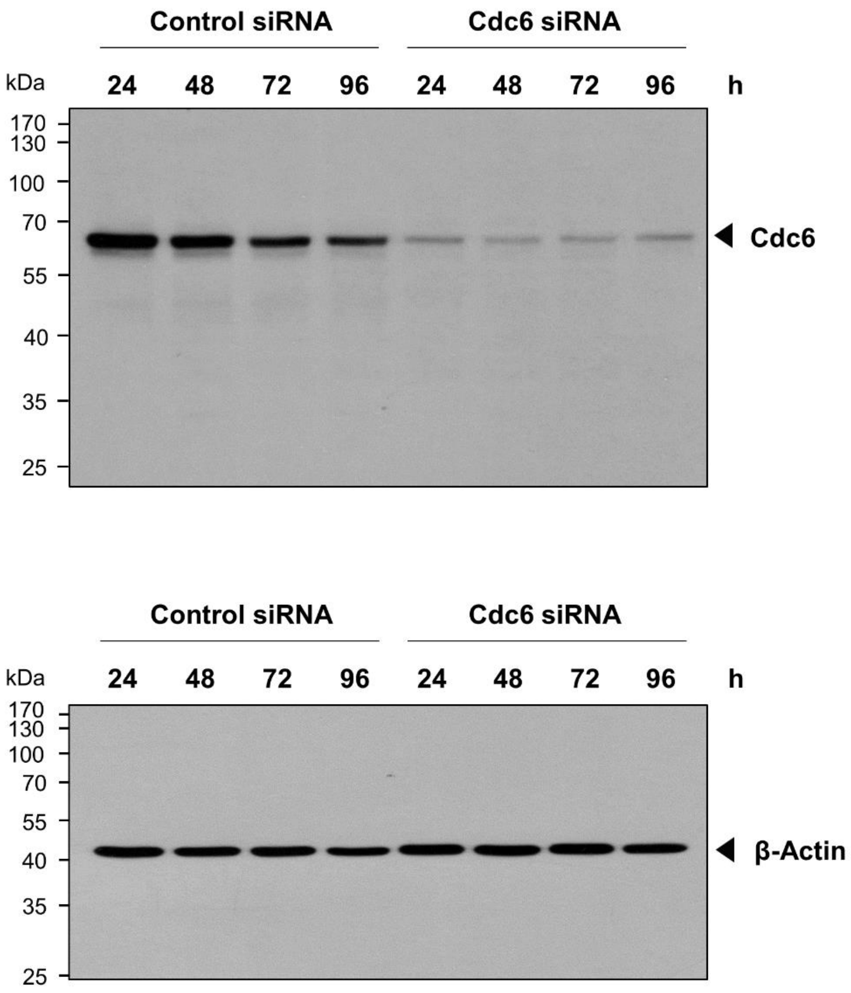

Supplementary Figure 3. Full-length immunoblots.

Figure 2. E

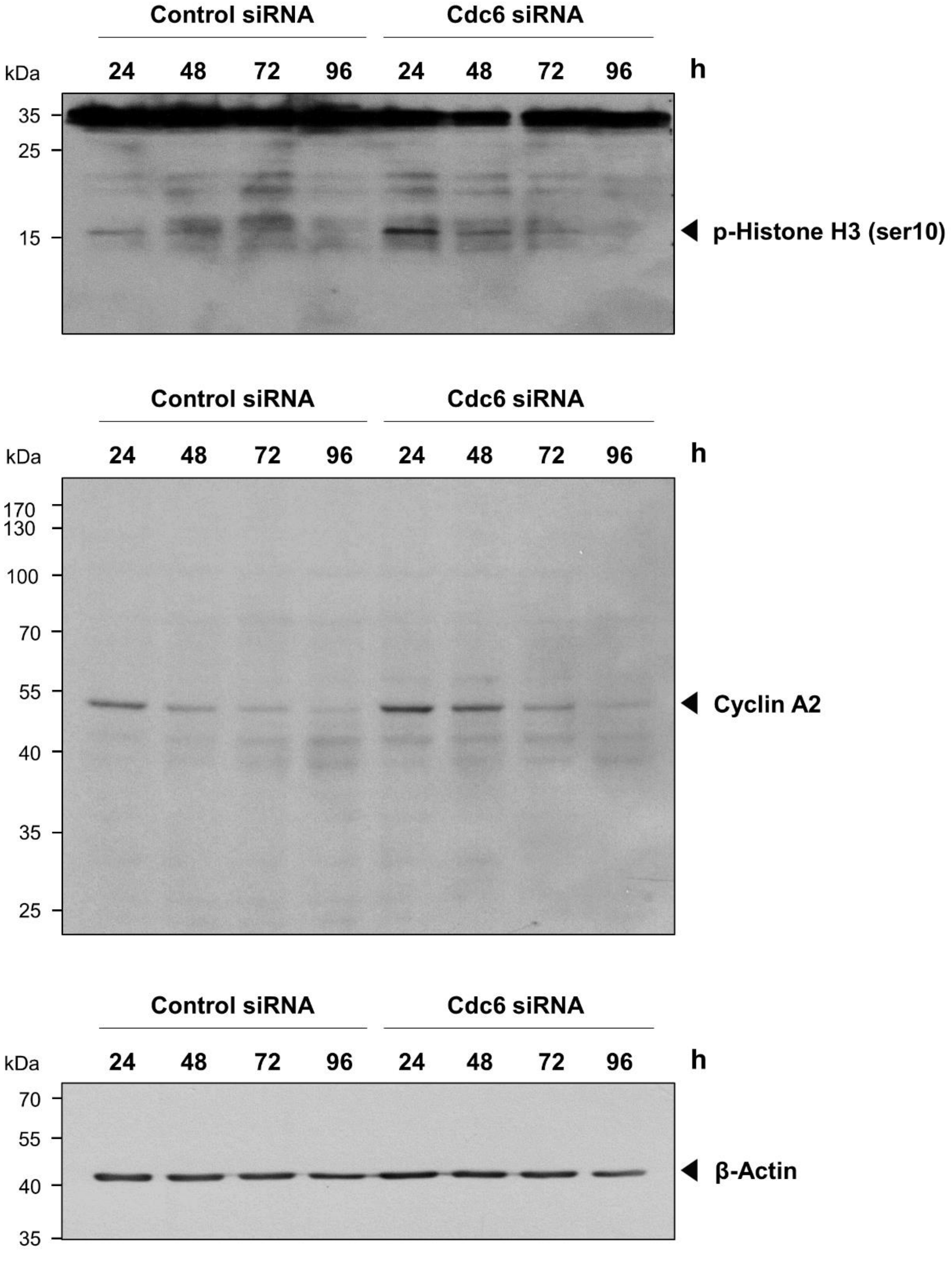

Supplementary Figure 3. Full-length immunoblots.

Figure 3. C

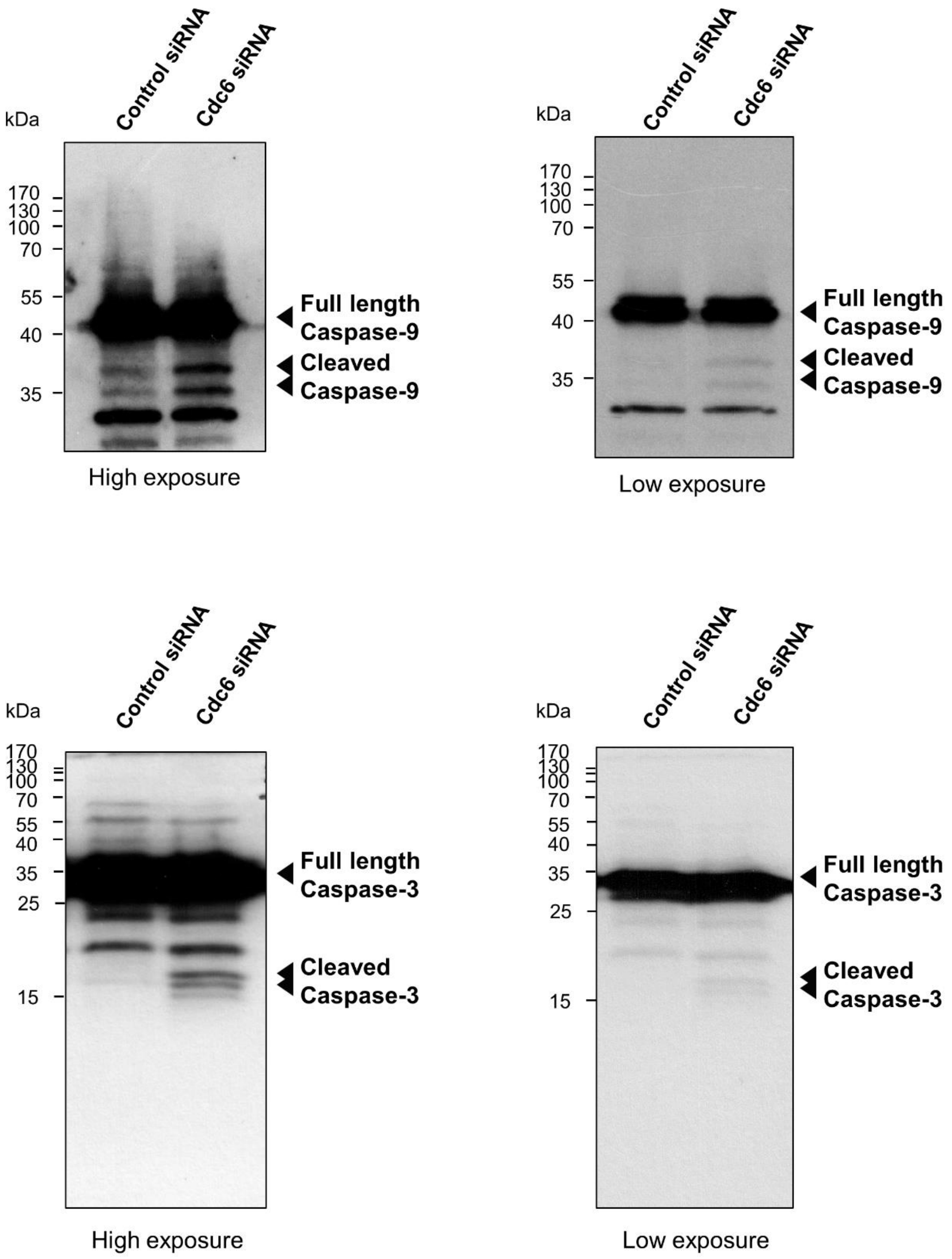

Supplementary Figure 3. Full-length immunoblots.

Figure 3. C

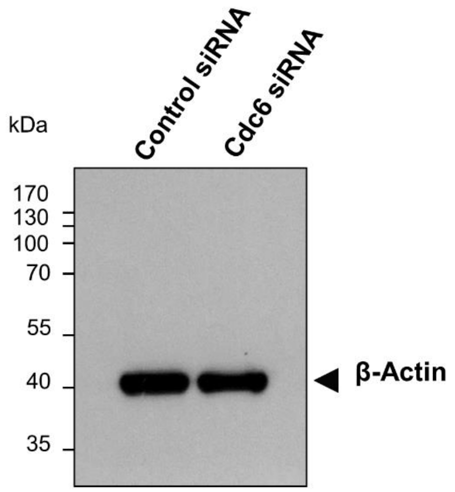

Supplementary Figure 1. A

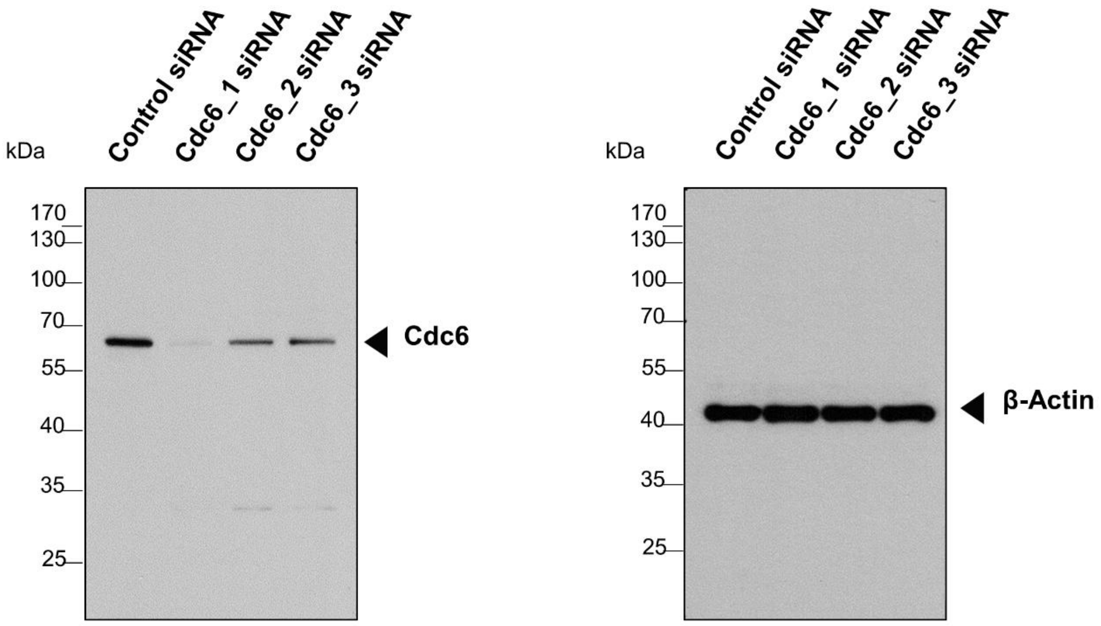

Supplementary Figure 3. Full-length immunoblots.
